# Metabotropic regulation of extrasynaptic GABA_A_ receptors

**DOI:** 10.3389/fncir.2013.00171

**Published:** 2013-10-25

**Authors:** William M. Connelly, Adam C. Errington, Giuseppe Di Giovanni, Vincenzo Crunelli

**Affiliations:** ^1^Neuroscience Division, Cardiff School of Biosciences, Cardiff UniversityCardiff, UK; ^2^Department of Physiology and Biochemistry, Faculty of Medicine, Malta UniversityMsida, Malta

**Keywords:** extrasynaptic, GABA, kinase, tonic, plasticity

## Abstract

A large body of work now shows the importance of GABA_A_ receptor-mediated tonic inhibition in regulating CNS function. However, outside of pathological conditions, there is relatively little evidence that the magnitude of tonic inhibition is itself under regulation. Here we review the mechanisms by which tonic inhibition is known to be modulated, and outline the potential behavioral consequences of this modulation. Specifically, we address the ability of protein kinase A and C to phosphorylate the extrasynaptic receptors responsible for the tonic GABA_A_ current, and how G-protein coupled receptors can regulate tonic inhibition through these effectors. We then speculate about the possible functional consequences of regulating the magnitude of the tonic GABA_A_ current.

## INTRODUCTION

GABA is the major inhibitory neurotransmitter in the mammalian forebrain. It is estimated that a third of synapses in the forebrain use GABA as their neurotransmitter (Bloom and Iversen, 1971). Through ionotropic GABA_A_ receptors, GABA works to increase membrane permeability to Cl^-^ (and to a lesser extent HCO_3_^-^) thereby reducing membrane impedance and potentially hyperpolarizing the membrane potential. The role of GABA_A_ receptor-mediated inhibition in the control of neural function is undeniable, and can be seen in nearly every aspect of neural function (Macdonald and Olsen, 1994; Freund and Buzsáki, 1996; Farrant and Nusser, 2005).

GABA_A_ receptors are believed to form as a pentameric assembly, out of 19 possible subunits (α1–6, β1–3, γ1–3, δ, ε, θ, π and ρ1–3), generally as a combination of α, β and γ subunits. Other combinations exist, where δ, ε, θ or π subunits replace the γ subunit. Finally, other permutations have been described, such as ρ homopentamers and receptors containing solely α and β subunits (Sieghart and Sperk, 2002). Importantly, different subunit combinations give GABA_A_ receptors different functional properties, e.g., different activation, deactivation and desensitization rates and altering their affinity for GABA and exogenous compounds (Verdoorn et al., 1990; Sigel et al., 1991). Furthermore, specific subunit combinations have specific expression patterns, often being expressed in restricted brain nuclei or neuronal cell types (Sieghart and Sperk, 2002). Finally, even on the level of a single cell, GABA_A_ receptors with a specific subunit make-up can be expressed in different subcellular compartments.

With this complexity in mind, a wealth of evidence has demonstrated that GABA_A_ receptors with specific subunit compositions, which are expressed in a unique spatial distribution, mediate a persistence or “tonic” inhibitory conductance. These receptors are generally α4βδ and α6βδ (though there are also α5βγ and others). They are expressed at a high density in the extrasynaptic compartment of dentate gyrus granule cells, cerebellar granule cells and thalamocortical cells (and to a lesser extent in olfactory bulb granule cells and striatal medium spiny cells) (Brickley and Mody, 2012). Due to their high affinity for GABA, and relatively slow desensitization rates, these extrasynaptic GABA_A_ receptors are believed to sense the activity dependent spill over of GABA from the synaptic cleft as well as the ambient concentration of GABA (and potentially they provide tonic inhibition in the absence of GABA; Wlodarczyk et al., 2013). There is a growing body of evidence showing the importance of tonic inhibition in regulating a variety of CNS functions, including sensory processing, controlling epileptiform activity and modulating anxiety states (Chadderton et al., 2004; Maguire et al., 2005; Cope et al., 2009). However, what is less clear is when and how the nature and magnitude of the tonic current are regulated. There are several studies that show that the magnitude of tonic current is altered in pathophysiological states, especially as a result of epilepsy, but it is less clear whether tonic currents are regulated during normal CNS function (Naylor et al., 2005; Payne et al., 2006; Zhang et al., 2007). Therefore, in this review, we will cover mechanisms by which tonic GABA_A_ inhibition can be regulated, specifically focusing on metabotropic regulation. Furthermore, we highlight potential paradigms where this regulation may be used *in vivo* to modulate inhibitory tone.

## KINASES

Phosphorylation is one of the most well understood post-translational modifications a protein can undergo. This reaction is catalyzed by kinases, and involves the transfer of a phosphate group from ATP to a serine, threonine or tyrosine residue in the target polypeptide. This phorphorylation changes the structure of the protein, and potentially its function. Due to the residues they target, kinases are generally subdivided into serine/threonine kinases such as calcium-dependent protein kinase (PKC) or cyclic AMP dependent protein kinase (PKA), tyrosine kinases such a v-Src, dual specificity kinases and histidine kinases (Edelman et al., 1987; Dhanasekaran and Premkumar Reddy, 1998; Schlessinger, 2000; West and Stock, 2001). Furthermore, while these families of kinases target a specific residue (or two, in the case of serine/threonine kinases), each individual family of kinases recognizes a general sequence of amino acid residues: a so called “consensus site.” This consensus site is in the order of 5–10 residues long, and is more or less specific depending on the family of kinases, for example, PKC is known for having a broad substrate specificity (Edelman et al., 1987). However, just because a protein contains a consensus site for a kinase, it does not guarantee that protein is a target for the kinase, for instance steric hindrance may prevent the kinase from accessing the site (Edelman et al., 1987).

## PKC MEDIATED REGULATION

**FIGURE 1:**
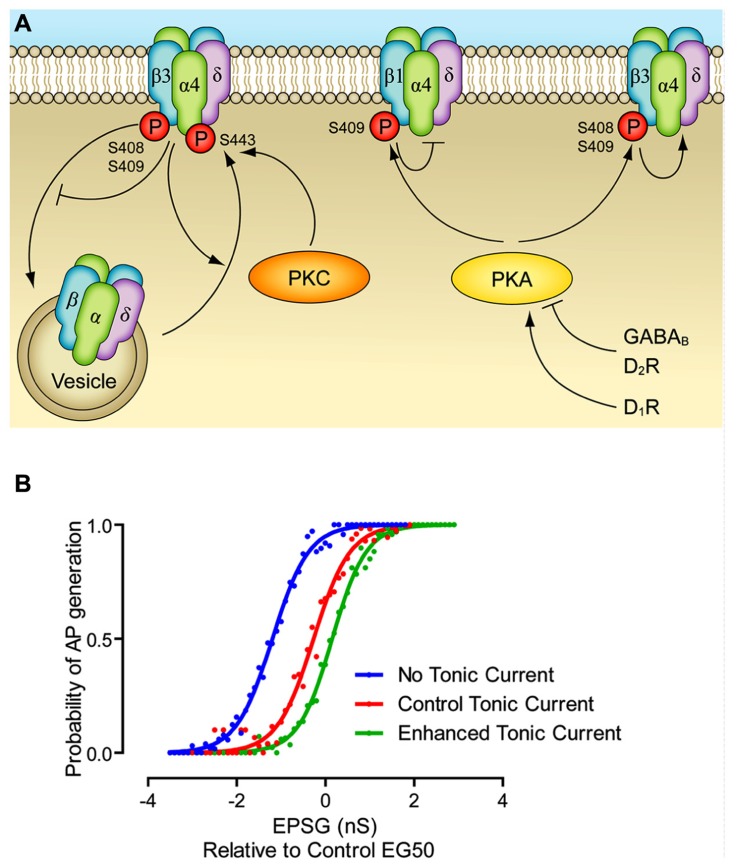
**Modulation of extrasynaptic GABA_A_ receptors. (A)** PKC leads to phosphorylation of α4 and β3 subunits, which increases cell surface stability. PKA leads to phosphorylation of β1 and β3 subunits, which inhibit and enhance GABA_A_ function, respectively. PKA mediated modulation of extrasynaptic currents has been demonstrated via dopamine D1 and D2 receptors, and via GABA_B_ receptors. Other potential pathways have been demonstrated, but they are not included here since they have not been as fully elucidated. Enhancement of a pathway is represented by an arrow head, inhibition is represented by a bar. **(B)** The effect of dynamic modulation of the tonic current on the neuronal input/output (I/O) function. Dynamic clamp was used to inject excitatory postsynaptic conductances (EPSGs) into neurons in control conditions, when the tonic GABA_A_ current was blocked by gabazine, and when the tonic current was enhanced to the level produced by GABA_B_ receptor agonists. There was no artificial injection of excitatory noise in this experiment, though the tonic current was produced via a noisy conductance model. Note that altering the level of the tonic current changes the offset of I/O function, without affecting the gain. Modified from Connelly et al. (2013).

One of the earliest pieces of evidence that GABA_A_ receptors can be modulated by kinases directly was provided by Sigel et al. (1991), who demonstrated that phorbol myristate acetate (PMA) stereo-selectively reduced the amplitude of evoked GABA currents recorded in Xenopus oocytes expressing GABA_A_ receptors with a variety of subunit compositions. Soon afterward, this effect was shown to be mediated by phosphorylation of both β and γ subunits, with serine 409 (S409) being the target on the β1 and β3 subunits and S410 being the target on β2 subunits, while S327 and S343 are the target on the γ2 subunit (**Figure 1**) (Kellenberger et al., 1992; Moss et al., 1992a; Krishek et al., 1994; McDonald and Moss, 1997). It is also worth noting that the alternative splicing that occurs on the γ2 subunit, which inserts 8 additional amino acids to create the γ2L subunit, adds a serine residue that satisfies the consensus site for phosphorylation by PKC and other kinases (Moss and Smart, 1996). Similarly, the β2 subunit is subjected to alternative splicing, though only in the chicken and human, and not rodent (McKinley et al., 1995). The β2L subunit is differentiated from the β2S subunit by an insertion of 17 amino acids in the chicken, and 38 amino acids in the human, both of which contain a strong consensus site for PKC (Harvey et al., 1994; McKinley et al., 1995). The α4 subunit appears to be unique amongst α subunits in that it expresses a consensus site between transmembrane domains 3 and 4 at S443 (**Figure 1**; Abramian et al., 2010). The recruitment of PKC to GABA_A_ receptors (and especially their β subunits) appears to be facilitated by the receptor for activated C kinase (RACK-1; Brandon et al., 1999).

The effect of PKC activation on GABA_A_ receptors is diverse, and appears to be dependent on the subunit composition in question. For instance, in hippocampal pyramidal cells, PKC appeared to have no effect on miniature inhibitory postsynaptic potentials (mIPSCs), while in dentate gyrus granule cells, PKC enhanced mIPSC amplitudes (Poisbeau et al., 1999). Furthermore, it has been shown that PKC causes an enhancement of receptor function in α1β1γ2L expressing cell lines (Lin et al., 1996) and an increase in mIPSC amplitudes mediated by αxβ3yx receptors (Jovanovic et al., 2004). Similarly, there is a large amount of evidence suggesting that PKC regulates the cell-surface expression and the stability at the membrane of GABA_A_ receptors. In both expression systems expressing α1β2γ2 and cultured cortical neurons, where there is constitutive recycling of GABA_A_ receptors from the cell-surface, PKC activity leads to a decrease of cell-surface GABA_A_ receptors and associated currents (Connolly et al., 1999; Filippova et al., 2000; Balduzzi et al., 2002; Herring et al., 2005). Interestingly, this effect appears to be independent of direct phosphorylation of the GABA_A_ receptor, and instead must involve phosphorylation of some other protein in the endocytotic cascade (Connolly et al., 1999). Thus, it is clear that synaptic GABA_A_ receptors can be modulated by PKC. Perhaps then it is surprising that there is such a paucity of results linking kinase action to the tonic GABA_A_ current. The following review of the available findings clearly indictes that further research into this area is warranted.

There is significant evidence that ethanol is a high affinity positive modulator of the α4/6βxδ receptors responsible for the tonic GABA_A_ current. Furthermore, this potentiation is, at least in part, responsible for the behavioral action of ethanol (Hanchar et al., 2005; Mody et al., 2007). Curiously, it appears as if the action of ethanol at these receptors is dependent on PKC. Choi et al. (2008) demonstrated both an anatomical and biochemical linkage between the PKC isozyme PKCδ and the δ subunit of the GABA_A _receptor. They reported that the distribution of PKCδ protein overlapped with that of the δ subunit. They went on to show that ethanol failed to potentiate tonic currents recorded from PKCδ knockout animals. Likewise, ethanol only potentiated α4β3δ mediated currents in cell lines also expressing PKCδ. Curiously, knocking out PKCδ appeared to have no effect on the baseline magnitude of the tonic current, indicating that at least in this paradigm, PKCδ only regulated the activity of other drugs at extrasynaptic receptors, rather than the activity of the receptors themselves. These effects are relatively rapid, and likely reflect the direct interaction of ethanol with the GABA_A_ receptor. It is worth noting that a similar effect has been observed for synaptic γ-containing GABA_A_ receptors and neurosteroids (e.g., Fáncsik et al., 2000). However, a negative interaction between kinase activity and neurosteroid action has also been noted at extrasynaptic receptors. In rats that had kindling-induced seizures, Kia et al. (2011) demonstrated that while the tonic current in CA1 pyramidal cells (likely mediated by α5β3γx) was similar to sham-controls, the extrasynaptic receptors were completely insensitive to the neurosteroid THDOC. This neurosteroid insensitivity could be reproduced in naive animals by PKC activation, though it is worth noting that the phosphatase activator Li-palmitate could not cause the tonic current recorded in kindled rats to show its normal THDOC sensitivity.

Just as kinase activity appears to regulate cell-surface expression of synaptic GABA_A_ receptors, there is evidence that kinases play a similar role at extrasynaptic receptors. Abramian et al. (2010) demonstrated that PKC activity in expression systems and hippocampal slices leads to an increase in α4 phosphorylation and cell surface expression, apparently at odds with what occurs at synaptic GABA_A_ receptors (e.g., Connolly et al., 1999). This increase in cell-surface expression was mirrored by an increase in GABA_A_ receptor mediated currents. Importantly, PKC activators could no longer enhance surface expression or currents in cell lines expressing a point mutation on the α4 subunit, whereby the phosphor-sensitive S443 residue was replaced with an alanine. These results contradict the analogous results seen at synaptic receptors, where PKC activity decreases cell surface expression, and is thought to do so independently of direct phosphorylation of GABA_A_ receptors (Connolly et al., 1999). PKC also seems to be able to regulate cell surface expression via phosphorylation of the β subunit, though apparently in an opposite direction to α4-phosphorylation reported by Abramian et al. (2010). Application of the PKC activator PMA inhibited tonic currents in dentate gyrus granule cells and thalamocortical cells and inhibiting PKC with bisindolylmaleimide I enhanced the tonic current (Bright and Smart, 2013). This result could be replicated in HEK293 cells expressing α4β2δ receptors, where the effect was dependent on phosphorylation at the S410 residue on β2 subunits, and independent of S443 on α4 subunits. Live cell imaging revealed that PKC activity was associated with a decrease in cell surface expression of δ-subunits (Bright and Smart, 2013). On the other hand, downstream of BDNF signaling, PKC has been shown to increase the cell surface stability of δ-subunits. BDNF was demonstrated to activate TrkB receptors, which in turn activated PLCγ. Presumably this then leads to an increase in intracellular Ca^2^^+^, and PKC activity, as the increased surface expression of δ-subunits was blocked by inhibitors of PLC and PKC. Unfortunately, the exact site of PKC phosphorylation on the GABA_A_ protein (or even whether it was on another protein altogether) was not elucidated (Joshi and Kapur, 2009).

## PKA MEDIATED REGULATION

PKA exists in many subtypes, but irrespective of the subtype, it is formed as a heterotetramer composed of two catyltic subunits held in an inactive state through an interaction with a dimer of regulatory subunits. PKA’s main regulatory mechanism is through binding of cAMP, but is also compartmentalized and regulated through an interaction with A-kinase-anchoring proteins (AKAPs; Pidoux and Taskén, 2010). PKA is a well established modulator of GABA_A_ receptors. While other subunits may contain PKA consensus sites, so far the only subunit that appears to be phosphoylated by PKA are the β subunits (McDonald et al., 1998 and citations therein). Indeed, more selectively than that, PKA appears to act only on β1 and β3 subunits, at S409 and S408/S409 respectively (Moss and Smart, 1996; McDonald et al., 1998). In HEK cells expressing α1β1γ2, PKA activation inhibits evoked GABA_A_ currents, while PKA enhances currents mediated by α1β3γ2 receptors (Moss et al., 1992b; McDonald et al., 1998). These results all come from synaptic subunit combinations.

However, the picture is not so clear cut for extrasynaptic isoforms. Tang et al. (2010) demonstrated that in HEK cells expressing α4β3δ receptors, PKA activation led to an increase in purely spontaneous GABA currents, that is, currents measured in the absence of GABA, while PKA had no effect on spontaneous currents measured from α4β3γ2L receptors. However, in the presence of low concentrations of GABA (1 μM), the effect was reversed, and PKA appeared to inhibit α4β3δ receptors. However, outside of expression systems, the effect of PKA becomes even more unclear. For instance, Poisbeau et al. (1999) reported that intracellular infusion of PKA suppressed mIPSCs recorded from hippocampal CA1 pyramidal cells, but had no effect on those recorded from dentate gyrus granule cells. This result cannot easily be explained in terms of differential expression of β subunits, as the both CA1 and dentate gyrus cells express all flavors of β subunit (Wisden et al., 1992). Likewise, while Nusser et al. (1999) reported that intracellular infusion of PKA enhanced mIPSC amplitude in olfactory granule cells (a cell type that only expresses the β3 subunit), Brünig et al. (1999) found that in the same cell type, dopamine D1 receptor agonists (which should stimulate adenylate cyclase and enhance PKA action) reduced evoked GABA_A_ receptor currents, in a manner that was blocked by PKA inhibitors, suggesting that PKA inhibits GABA_A_ receptors in these cells. It is possible to explain this result in light of the fact that Brünig et al. (1999) worked on cultured neurons, activating PKA through a more physiological G-protein coupled approach, while Nusser et al. (1999) used native tissue, but caused phosphorylation through infusing active PKA. Finally, it may also appear that the length of time PKA activity is increased for can produce different effects, as Angelotti et al. (1993) reported that expression of α1β1γ2S receptors in cell lines with higher PKA activity had higher GABA_A_ receptor mediated currents than those with lower PKA activity, in comparison to direct application of PKA activators that usually inhibit β1 containing GABA_A_ receptors.

Regarding PKA activity at extrasynaptic GABA_A_ receptors, there are two papers which appear to reveal the picture. Janssen et al. (2009) demonstrated that a dopamine D1 receptor agonist enhanced a tonic current believed to be mediated by α5β3γ receptors in D1-positive striatal medium spiny neurons, while a D2 receptor agonist (which should inhibit adenylate cyclase and inhibit PKA action) reduced the tonic current (believed to be mediated by the same α5β3γ) in D2-positive neurons. Curiously, PKA infusion enhanced the tonic current in D1-positive medium spiny neurons, while it inhibited the current in D2-positive neurons. Thus, while experiments involving dopamine receptor agonists support the notion of McDonald et al. (1998) that PKA activity at β3 containing receptors enhances GABA_A_ receptor function, the experiments involving PKA infusion paint a more complex picture. However, the results can be understood when one considers that the PKA inhibitor PKI reduced the tonic current in D2-positive cells, but had no effect in D1-positive cells, implying that β3 containing receptors are basally phosphorylated at D1-positive cells, but not at D2-positive cells. Thus application of PKA to D1-positive cells would have no action at β3 subunits, and may potentially be having its effect via a small proportion of β1 containing receptors. In a more straightforward to interpret result, Connelly et al. (2013) demonstrated that activating the GABA_B_ receptor enhanced the α4βδ mediated tonic current in thalamocortical cells and dentate gyrus granule cells, as well as the α6βδ mediated tonic current in cerebellar granule cells, an effect mimicked by PKA inhibitors. Inversely, infusing PKA decreased the tonic current, however the β subunit involvement was not determined in this paper (also see Tao et al., 2013).

PKA is also known to regulate the cell surface stability of GABA_A_ receptors. For instance, dopamine D3 receptor activation has been shown to increase the rate of clatherin-mediated endocytosis of synaptic GABA_A_ receptors in a PKA-dependent fashion (Chen et al., 2006). Likewise, PKA can regulate the expression of GABA_A_ receptors. Specifically, the expression of the δ subunit is known to be highly dynamic in cerebellar granule cells (e.g., Payne et al., 2008). Uusi-Oukari et al. (2010) reported that AMPA receptor activation led to an increase in δ-subunit mRNA in cultured cerebellar granule cells, and that this effect was dependent on PKA.

## OTHER KINASE MEDIATED REGULATION

There are only a small number of studies investigating the effects of non-PKA/PKC mediated modulation of the tonic GABA_A_ current, indicating the need for more research in this area. Tyrosine kinases can phosphorylate GABA_A_ γ2 subunits at Y365/367, which reduces the ability of the clathrin-adaptor protein, AP2, to bind, resulting in reduced internalization and the subsequent increase in membrane insertion of the channels (Moss et al., 1995; Kittler et al., 2008). It appears that this site is constitutively phorphorylated and its effect is more readily seen by blocking phosphorylation, rather than enhancing it (Brandon et al., 2001). Therefore Nani et al. (2013) used a Y365/367F mouse line, where the principle tyrosine sites were mutated to phenylalanine, blocking phosphorylation and AP2 binding. As would be predicted, spontaneous IPSC amplitude was increased, while the decay was unaffected. Curiously, the expression of α4 and δ subunits were increased, as were the tonic current recorded in dorsal lateral geniculate neurons. Furthermore, these effects seemed limited to female mice. Exactly how alterations in γ2 surface expression lead to an increase in α4 and δ subunit expression is unclear.

Ca^2^^+^/calmodulin-dependent protein kinase II (CaMKII) is a serine/threonine that has been demonstrated to be able to modulate synaptic inhibition in a wide variety of cell types (e.g., Wang et al., 1995). Saliba et al. (2012) extended these findings by showing that CaMKII activation, subsequent to Ca^2^^+^ influx produced by Bay K 8644 application, produced a profound increase in surface insertion of α5 and β3 subunits, and an increase in a tonic current mediated by α5β3γ2 receptors. This effect was mediated by phosphorylation at S383 on the β3 subunit. While in these experiments Ca2+ influx was caused by Bay K 8644 or 4-AP application, they do suggest the possibility of activity dependent regulation of the tonic current (see Implications).

Wang et al. (2012) investigated how acute systemic inflammation leads to memory loss. Systemic interleukin-1β (IL-1β) injections caused an impairment of contextual fear memory, an effect which was absent in α5^-^^/^^-^ animals or in animals treated with L-655, 708, an inverse agonist selective for α5-containin GABA_A_ receptors. Acute systemic IL-1β injections or *in vitro* application of IL-1β both caused an increase in the tonic current measured in hippocampal CA1 cells, where there was a concurrent increase in α5 subunit surface expression. This effect was dependent on the activity of serine/threonine kinase, p38 mitogen-activated protein kinase (MAPK), though how it causes increased cell-surface expression of α5 subunit containing receptors is still unclear.

## PRESYNAPTIC REGULATION OF TONIC CURRENT

As the δ-containing GABA_A_ receptors appear to sense ambient GABA and/or GABA which spills over from the synaptic cleft, it seems likely that manipulations that increase the release of GABA will increase the magnitude of the tonic current. Indeed, it appears that blocking action potential dependent release of GABA can reduce the size of the tonic current (e.g., Brickley et al., 1996; Glykys and Mody, 2007; though see Wall and Usowicz, 1997; Rossi et al., 2003). But can more subtle manipulations of GABA release alter the tonic current? Indeed, it appears that they can. Rossi et al. (2003) demonstrated that in cerebellar granule cells, acetylcholine, acting through nicotinic receptors, produces a largely vesicular, Ca^2^^+^ dependent, action potential-independent release of GABA that causes a 12 fold enhancement in the magnitude of the tonic GABA_A_ current. The exact source of this GABA is unclear, but the authors speculate that it is caused by presynaptic nicotinic receptors on interneuron terminals, causing presynaptic depolarization, and hence vesicular GABA release. This finding is mirrored by Errington et al. (2011) who reported that group I metabotropic glutamate receptor agonists cause an increase in spontaneous IPSC frequency and tonic current in thalamocortical neurons of the dorsal lateral geniculate nucleus. While the IPSCs are clearly action potential-dependent, the increase in tonic current was independent of action potentials, again pointing to the notion that presynaptic receptors were facilitating release from interneurons (for similar findings see Krishek et al., 1994; Kullmann and Semyanov, 2002). The inverse case was demonstrated by Bright and Brickley (2008), where depolarization of ventrobasal thalamocortical cells induced a robust increase in spontaneous IPSC frequency, but failed to affect the tonic current. Thus, presynaptic modulation of GABA release can enhance the tonic current, but increasing action potential-dependent release does not necessarily enhance the tonic current.

## IMPLICATIONS

If the tonic GABA_A_ current simply provides a hyperpolarizing/shunting influence on the membrane, why do neurons use it, rather than classical leak potassium channels? One suggestion is that the largely shunting inhibition provided by tonic inhibition alters the input/output function of the neuron in a way that hyperpolarizing inhibition (as produced by potassium channels) cannot. That is to say, hyperpolarizing inhibition alters the offset (the excitatory input needed to bring the cell to fire) while not greatly affecting the gain, i.e., the relationship between input excitation and firing rate. Shunting inhibition is often suggested to largely have the opposite effect, reducing the gain, while not affecting the offset. However, it appears that the situation is more complex, and also depends on the nature of the excitatory drive, specifically, during tonic excitation shunting inhibition affects only the offset, while during noisy trains of excitation shunting inhibition mainly alters the gains (**Figure 1B**; Holt and Koch, 1997; Mitchell and Silver, 2003; Prescott and Koninck, 2003; Semyanov et al., 2004). Indeed, this is further complicated by the rectifying property of the tonic current, as seen in several cell types (Pavlov et al., 2009; Ransom et al., 2010). We suggest a reason that may also come into play is the plasticity afforded to the tonic GABA_A_ receptor system. As described above (**Table 1**), there are a multitude of pathways by which the magnitude of the tonic current can be modulated, with most of them largely independent of synaptic GABA release. This means that, as opposed to regulation of the potassium channels responsible for the resting membrane potential, modulating tonic GABA_A_ inhibition affects neuronal excitability largely independently of the resting membrane potential.

**Table 1:** Summary of the effects of kinase action on GABA_A_ receptor mediated tonic currents.

Effector	Effect	Reference
PKC	Increased membrane insertion of α4 subunits	Abramian etal. (2010)
	Reduced surface expression of δ subunits due to β2 phosphorylation	Bright and Smart (2013)
PKA	Enhanced tonic current in D1+ medium spiny neurons. Reduced tonic current in D2+ medium spiny neurons.	Janssen etal. (2009) Connelly etal. (2013), Tao etal. (2013)
	Reduced tonic current in thalamocortical neurons, dentate gyrus granule cells and cerebellar granule cells.	
Tyrosine kinase	Reduced γ2 internalization, subsequently increases α4 and δ expression	Nani etal. (2013)
CaMKII	Increases insertion of α5 and β3 subunits	Saliba etal. (2012)
MAPK	Increased membrane insertion of α5 subunit	Wang etal. (2012)

The results cited above clearly demonstrate that the tonic GABA_A_ system is susceptible to modulation (**Figure 1**). While there have been some studies showing a role of dynamic modulation of the tonic current, for instance in response to ethanol abuse or in response to epilepsy, these effects are generally seen to be due to changes in expression (Cagetti et al., 2003; Maguire et al., 2005; Payne et al., 2007). It would be fascinating to investigate whether more rapid changes in the magnitude of tonic current can occur due to kinase-dependent modulation, for instance during the switch between different levels of vigilance (in response to changing levels of brain stem neuromodulators). On a simpler, cellular level, the results summarized above show the diversity of effects caused by kinase action on extrasynaptic GABA_A_ receptors. However, there are relatively few data demonstrating whether G-protein coupled receptors are able to induce the same effects. Similarly, while PKC has been shown to modulate extrasynaptic GABA_A_ receptors, we are not aware of any papers that show that interventions that cause a rise in intracellular Ca^2^^+^ (and hence PKC activity) can modulate the tonic current through PKC (though see Saliba et al., 2012). Hence, the notion of activity-dependent regulation of δ-containing GABA_A_ receptors remains attractive, yet elusive.

## Conflict of Interest Statement

The authors declare that the research was conducted in the absence of any commercial or financial relationships that could be construed as a potential conflict of interest.

## References

[B1] Abramian A. M. Comenencia-Ortiz E. Vithlani M. Tretter E. V. Sieghart W. Davies P. A. (2010). Protein kinase C phosphorylation regulates membrane insertion of GABAA receptor subtypes that mediate tonic inhibition. *J. Biol. Chem.* 285 41795–41805 10.1074/jbc.M110.149229 20940303 PMC3009907

[B2] Angelotti T. P. Uhler M. D. Macdonald R. L. (1993). Enhancement of recombinant gamma-aminobutyric acid type A receptor currents by chronic activation of cAMP-dependent protein kinase. *Mol. Pharmacol.* 44 1202–1210 8264557

[B3] Balduzzi R. Cupello A. Robello M. (2002). Modulation of the expression of GABA(A) receptors in rat cerebellar granule cells by protein tyrosine kinases and protein kinase C. *Biochim. Biophys. Acta* 1564 263–270 10.1016/S0005-2736(02)00460-1 12101021

[B4] Bloom F. E. Iversen L. L. (1971). Localizing 3H-GABA in nerve terminals of rat cerebral cortex by electron microscopic autoradiography. *Nature* 229 628–630 10.1038/229628a0 4925465

[B5] Brandon N. J. Delmas P. Hill J. Smart T. G. Moss S. J. (2001). Constitutive tyrosine phosphorylation of the GABAA receptor γ2 subunit in rat brain. *Neuropharmacology* 41 745–752 10.1016/S0028-3908(01)00121-6 11640929

[B6] Brandon N. J. Uren J. M. Kittler J. T. Wang H. Olsen R. Parker P. J. (1999). Subunit-specific association of protein kinase C and the receptor for activated C kinase with GABA type A receptors. *J. Neurosci.* 19 9228–9234 10531426 10.1523/JNEUROSCI.19-21-09228.1999 PMC6782933

[B7] Brickley S. G. Cull-Candy S. G. Farrant M. (1996). Development of a tonic form of synaptic inhibition in rat cerebellar granule cells resulting from persistent activation of GABAA receptors. *J. Physiol.* 497 753–759 9003560 10.1113/jphysiol.1996.sp021806 PMC1160971

[B8] Brickley S. G. Mody I. (2012). Extrasynaptic GABA(A) receptors: their function in the CNS and implications for disease. *Neuron* 73 23–34 10.1016/j.neuron.2011.12.012 22243744 PMC3399243

[B9] Bright D. P. Brickley S. G. (2008). Acting locally but sensing globally: impact of GABAergic synaptic plasticity on phasic and tonic inhibition in the thalamus. *J. Physiol.* 586 5091–5099 10.1113/jphysiol.2008.158576 18772202 PMC2652165

[B10] Bright D. P. Smart T. G. (2013). Protein kinase C regulates tonic GABA_A_ receptor-mediated inhibition in the hippocampus and thalamus. *Eur. J. Neurosci.* 10.1111/ejn.12352 [Epub ahead of print] PMC4165308 24102973

[B11] Brünig I. Sommer M. Hatt H. Bormann J. (1999). Dopamine receptor subtypes modulate olfactory bulb gamma-aminobutyric acid type A receptors. *Proc. Natl. Acad. Sci. U.S.A.* 96 2456–2460 10.1073/pnas.96.5.2456 10051664 PMC26806

[B12] Cagetti E. Liang J. Spigelman I. Olsen R. W. (2003). Withdrawal from chronic intermittent ethanol treatment changes subunit composition, reduces synaptic function, and decreases behavioral responses to positive allosteric modulators of GABAA receptors. *Mol. Pharmacol.* 63 53–64 10.1124/mol.63.1.53 12488536

[B13] Chadderton P. Margrie T. W. Häusser M. (2004). Integration of quanta in cerebellar granule cells during sensory processing. *Nature* 428 856–860 10.1038/nature02442 15103377

[B14] Chen G. Kittler J. T. Moss S. J. Yan Z. (2006). Dopamine D3 receptors regulate GABAA receptor function through a phospho-dependent endocytosis mechanism in nucleus accumbens. *J. Neurosci.* 26 2513–2521 10.1523/JNEUROSCI.4712-05.2006 16510729 PMC6793654

[B15] Choi D.-S. Wei W. Deitchman J. K. Kharazia V. N. Lesscher H. M. B. McMahon T. (2008). Protein kinase Cdelta regulates ethanol intoxication and enhancement of GABA-stimulated tonic current. *J. Neurosci.* 28 11890–11899 10.1523/JNEUROSCI.3156-08.2008 19005054 PMC2688726

[B16] Connelly W. M. Fyson S. J. Errington A. C. McCafferty C. P. Cope D. W. Di Giovanni G. (2013). GABAB receptors regulate extrasynaptic GABAA receptors. *J. Neurosci.* 33 3780–3785 10.1523/JNEUROSCI.4989-12.2013 23447590 PMC3601669

[B17] Connolly C. N. Kittler J. T. Thomas P. Uren J. M. Brandon N. J. Smart T. G. (1999). Cell surface stability of gamma-aminobutyric acid type A receptors. Dependence on protein kinase C activity and subunit composition. *J. Biol. Chem.* 274 36565–36572 10.1074/jbc.274.51.36565 10593956

[B18] Cope D. W. Di Giovanni G. Fyson S. J. Orbán G. Errington A. C. Lorincz M. L. (2009). Enhanced tonic GABAA inhibition in typical absence epilepsy. *Nat. Med.* 15 1392–1398 10.1038/nm.2058 19966779 PMC2824149

[B19] Dhanasekaran N Premkumar Reddy E. (1998). Signaling by dual specificity kinases. *Oncogene* 17 1447–1455 10.1038/sj.onc.1202251 9779990

[B20] Edelman A. M. Blumenthal D. K. Krebs E. G. (1987). Protein serine/threonine kinases. *Annu. Rev. Biochem.* 56 567–613 10.1146/annurev.bi.56.070187.003031 2956925

[B21] Errington A. C. Di Giovanni G. Crunelli V. Cope D. W. (2011). mGluR control of interneuron output regulates feedforward tonic GABAA inhibition in the visual thalamus. *J. Neurosci.* 31 8669–8680 10.1523/JNEUROSCI.0317-11.2011 21653871 PMC3130900

[B22] Fáncsik A. Linn D. M. Tasker J. G. (2000). Neurosteroid modulation of GABA IPSCs is phosphorylation dependent. *J. Neurosci.* 20 3067–3075 10777770 10.1523/JNEUROSCI.20-09-03067.2000 PMC6773128

[B23] Farrant M. Nusser Z. (2005). Variations on an inhibitory theme: phasic and tonic activation of GABA(A) receptors. *Nat. Rev. Neurosci.* 6 215–229 10.1038/nrn1625 15738957

[B24] Filippova N. Sedelnikova A. Zong Y. Fortinberry H. Weiss D. S. (2000). Regulation of recombinant gamma-aminobutyric acid (GABA)(A) and GABA(C) receptors by protein kinase C. *Mol. Pharmacol.* 57 847–856 10779366

[B25] Freund T. F. Buzsáki G. (1996). Interneurons of the hippocampus. *Hippocampus* 6 347–470 10.1002/(SICI)1098-1063(1996)6:4<347::AID-HIPO1>3.0.CO;2-I 8915675

[B26] Glykys J. Mody I. (2007). The main source of ambient GABA responsible for tonic inhibition in the mouse hippocampus. *J. Physiol.* 582 1163–1178 10.1113/jphysiol.2007.134460 17525114 PMC2075237

[B27] Hanchar H. J. Dodson P. D. Olsen R. W. Otis T. S. Wallner M. (2005). Alcohol-induced motor impairment caused by increased extrasynaptic GABA(A) receptor activity. *Nat. Neurosci.* 8 339–345 10.1038/nn1398 15696164 PMC2854077

[B28] Harvey R. J. Chinchetru M. A. Darlison M. G. (1994). Alternative splicing of a 51-nucleotide exon that encodes a putative protein kinase C phosphorylation site generates two forms of the chicken gamma-aminobutyric acidA receptor beta 2 subunit. *J. Neurochem.* 62 10–16 10.1046/j.1471-4159.1994.62010010.x 7505310

[B29] Herring D. Huang R. Singh M. Dillon G. H. Leidenheimer N. J. (2005). PKC modulation of GABAA receptor endocytosis and function is inhibited by mutation of a dileucine motif within the receptor β2 subunit. *Neuropharmacology* 48 181–194 10.1016/j.neuropharm.2004.09.015 15695157

[B30] Holt G. R. Koch C. (1997). Shunting inhibition does not have a divisive effect on firing rates. *Neural Comput.* 9 1001–1013 10.1162/neco.1997.9.5.1001 9188191

[B31] Janssen M. J. Ade K. K. Fu Z. Vicini S. (2009). Dopamine modulation of GABA tonic conductance in striatal output neurons. *J. Neurosci.* 29 5116–5126 10.1523/JNEUROSCI.4737-08.2009 19386907 PMC2707274

[B32] Joshi S. Kapur J. (2009). Slow intracellular accumulation of GABA(A) receptor delta subunit is modulated by brain-derived neurotrophic factor. *Neuroscience* 164 507–519 10.1016/j.neuroscience.2009.08.008 19665523 PMC2761981

[B33] Jovanovic J. N. Thomas P. Kittler J. T. Smart T. G. Moss S. J. (2004). Brain-derived neurotrophic factor modulates fast synaptic inhibition by regulating GABA(A) receptor phosphorylation, activity, and cell-surface stability. *J. Neurosci.* 24 522–530 10.1523/JNEUROSCI.3606-03.2004 14724252 PMC6729993

[B34] Kellenberger S. Malherbe P. Sigel E. (1992). Function of the alpha 1 beta 2 gamma 2S gamma-aminobutyric acid type A receptor is modulated by protein kinase C via multiple phosphorylation sites. *J. Biol. Chem.* 267 25660–25663 1334482

[B35] Kia A. Ribeiro F. Nelson R. Gavrilovici C. Ferguson S. S. G. Poulter M. O. (2011). Kindling alters neurosteroid-induced modulation of phasic and tonic GABAA receptor-mediated currents: role of phosphorylation. *J. Neurochem.* 116 1043–1056 10.1111/j.1471-4159.2010.07156.x 21175618

[B36] Kittler J. T. Chen G. Kukhtina V. Vahedi-Faridi A. Gu Z. Tretter V. (2008). Regulation of synaptic inhibition by phospho-dependent binding of the AP2 complex to a YECL motif in the GABAA receptor γ2 subunit. *PNAS* 105 3616–3621 10.1073/pnas.0707920105 18305175 PMC2265186

[B37] Krishek B. J. Xie X. Blackstone C. Huganir R. L. Moss S. J. Smart T. G. (1994). Regulation of GABAA receptor function by protein kinase C phosphorylation. *Neuron* 12 1081–1095 10.1016/0896-6273(94)90316-6 8185945

[B38] Kullmann D. M. Semyanov A. (2002). Glutamatergic modulation of GABAergic signaling among hippocampal interneurons: novel mechanisms regulating hippocampal excitability. *Epilepsia* 43 174–178 10.1046/j.1528-1157.43.s.5.12.x 12121316

[B39] Lin Y. F. Angelotti T. P. Dudek E. M. Browning M. D. Macdonald R. L. (1996). Enhancement of recombinant alpha 1 beta 1 gamma 2L gamma-aminobutyric acidA receptor whole-cell currents by protein kinase C is mediated through phosphorylation of both beta 1 and gamma 2L subunits. *Mol. Pharmacol.* 50 185–195 8700112

[B40] Macdonald R. L. Olsen R. W. (1994). GABAA receptor channels. *Annu. Rev. Neurosci.* 17 569–602 10.1146/annurev.ne.17.030194.003033 7516126

[B41] Maguire J. L. Stell B. M. Rafizadeh M. Mody I. (2005). Ovarian cycle-linked changes in GABA(A) receptors mediating tonic inhibition alter seizure susceptibility and anxiety. *Nat. Neurosci.* 8 797–804 10.1038/nn1469 15895085

[B42] McDonald B. J. Amato A. Connolly C. N. Benke D. Moss S. J. Smart T. G. (1998). Adjacent phosphorylation sites on GABAA receptor β subunits determine regulation by cAMP-dependent protein kinase. *Nat. Neurosci.* 1 23–28 10.1038/223 10195104

[B43] McDonald B. J. Moss S. J. (1997). Conserved phosphorylation of the intracellular domains of GABA(A) receptor beta2 and beta3 subunits by cAMP-dependent protein kinase, cGMP-dependent protein kinase protein kinase C and Ca2+/calmodulin type II-dependent protein kinase. *Neuropharmacology* 36 1377–1385 10.1016/S0028-3908(97)00111-1 9423925

[B44] McKinley D. D. Lennon D. J. Carter D. B. (1995). Cloning, sequence analysis and expression of two forms of mRNA coding for the human beta 2 subunit of the GABAA receptor. *Brain Res. Mol. Brain Res.* 28 175–179 10.1016/0169-328X(94)00228-7 7707873

[B45] Mitchell S. J. Silver R. A. (2003). Shunting inhibition modulates neuronal gain during synaptic excitation. *Neuron* 38 433–445 10.1016/S0896-6273(03)00200-9 12741990

[B46] Mody I. Glykys J. Wei W. (2007). A new meaning for “Gin & Tonic”: tonic inhibition as the target for ethanol action in the brain. *Alcohol* 41 145–153 10.1016/j.alcohol.2007.03.009 17521846 PMC2012942

[B47] Moss S. J. Doherty C. A. Huganir R. L. (1992a). Identification of the cAMP-dependent protein kinase and protein kinase C phosphorylation sites within the major intracellular domains of the beta 1, gamma 2S, and gamma 2L subunits of the gamma-aminobutyric acid type A receptor. *J. Biol. Chem.* 267 14470–14476 1321150

[B48] Moss S. J. Smart T. G. Blackstone C. D. Huganir R. L. (1992b). Functional modulation of GABAA receptors by cAMP-dependent protein phosphorylation. *Science* 257 661–665 10.1126/science.1323140 1323140

[B49] Moss S. J. Gorrie G. H. Amato A. Smart T. G. (1995). Modulation of GABAA receptors by tyrosine phosphorylation. *Nature* 377 344–348 10.1038/377344a0 7566089

[B50] Moss S. J. Smart T. G. (1996). Modulation of amino acid-gated ion channels by protein phosphorylation. *Int. Rev. Neurobiol.* 39 1–52 10.1016/S0074-7742(08)60662-5 8894843

[B51] Nani F. Bright D. P. Revilla-Sanchez R. Tretter V. Moss S. J. Smart T. G. (2013). Tyrosine phosphorylation of GABAA receptor γ2-subunit regulates tonic and phasic inhibition in the thalamus. *J. Neurosci.* 33 12718–12727 10.1523/JNEUROSCI.0388-13.2013 23904608 PMC4400286

[B52] Naylor D. E. Liu H. Wasterlain C. G. (2005). Trafficking of GABA(A) receptors, loss of inhibition, and a mechanism for pharmacoresistance in status epilepticus. *J. Neurosci.* 25 7724–7733 10.1523/JNEUROSCI.4944-04.2005 16120773 PMC6725248

[B53] Nusser Z. Sieghart W. Mody I. (1999). Differential regulation of synaptic GABAA receptors by cAMP-dependent protein kinase in mouse cerebellar and olfactory bulb neurones. *J. Physiol. (Lond.) *521(Pt 2) 421–435 10.1111/j.1469-7793.1999.00421.x PMC2269679 10581313

[B54] Pavlov I. Savtchenko L. P. Kullmann D. M. Semyanov A. Walker M. C. (2009). Outwardly rectifying tonically active GABAA receptors in pyramidal cells modulate neuronal offset, not gain. *J. Neurosci.* 29 15341–15350 10.1523/JNEUROSCI.2747-09.2009 19955387 PMC6665960

[B55] Payne H. L. Connelly W. M. Ives J. H. Lehner R. Furtmuller B. Sieghart W. (2007). GABAA alpha6-containing receptors are selectively compromised in cerebellar granule cells of the ataxic mouse, stargazer. *J. Biol. Chem.* 282 29130–29143 10.1074/jbc.M700111200 17646167 PMC2974090

[B56] Payne H. L. Donoghue P. S. Connelly W. M. K. Hinterreiter S. Tiwari P. Ives J. H. (2006). Aberrant GABA(A) receptor expression in the dentate gyrus of the epileptic mutant mouse stargazer. *J. Neurosci.* 26 8600–8608 10.1523/JNEUROSCI.1088-06.2006 16914686 PMC2974089

[B57] Payne H. L. Ives J. H. Sieghart W. Thompson C. L. (2008). AMPA and kainate receptors mediate mutually exclusive effects on GABA(A) receptor expression in cultured mouse cerebellar granule neurones. *J. Neurochem.* 104 173–186 10.1111/j.1471-4159 17986225

[B58] Pidoux G. Taskén K. (2010). Specificity and spatial dynamics of protein kinase A signaling organized by A-kinase-anchoring proteins. *J. Mol. Endocrinol.* 44 271–284 10.1677/JME-10-0010 20150326

[B59] Poisbeau P. Cheney M. C. Browning M. D. Mody I. (1999). Modulation of synaptic GABAA receptor function by PKA and PKC in adult hippocampal neurons. *J. Neurosci.* 19 674–683 9880588 10.1523/JNEUROSCI.19-02-00674.1999 PMC6782188

[B60] Prescott S. A. Koninck Y. D. (2003). Gain control of firing rate by shunting inhibition: roles of synaptic noise and dendritic saturation. *PNAS* 100 2076–2081 10.1073/pnas.0337591100 12569169 PMC149961

[B61] Ransom C. B. Wu Y. Richerson G. B. (2010). Postdepolarization potentiation of GABAA receptors: a novel mechanism regulating tonic conductance in hippocampal neurons. *J. Neurosci.* 30 7672–7684 10.1523/JNEUROSCI.0290-10.2010 20519542 PMC2902370

[B62] Rossi D. J. Hamann M. Attwell D. (2003). Multiple modes of GABAergic inhibition of rat cerebellar granule cells. *J. Physiol.* 548 97–110 10.1113/jphysiol.2002.036459 12588900 PMC2342786

[B63] Saliba R. S. Kretschmannova K. Moss S. J. (2012). Activity-dependent phosphorylation of GABAA receptors regulates receptor insertion and tonic current. *EMBO J.* 31 2937–2951 10.1038/emboj.2012.109 22531784 PMC3395084

[B64] Schlessinger J. (2000). Cell signaling by receptor tyrosine kinases. *Cell* 103 211–225 10.1016/S0092-8674(00)00114-8 11057895

[B65] Semyanov A. Walker M. C. Kullmann D. M. Silver R. A. (2004). Tonically active GABAA receptors: modulating gain and maintaining the tone. *Trends Neurosci.* 27 262–269 10.1016/j.tins.2004.03.005 15111008

[B66] Sieghart W. Sperk G. (2002). Subunit composition, distribution and function of GABA(A) receptor subtypes. *Curr. Top. Med. Chem* 2 795–816 10.2174/1568026023393507 12171572

[B67] Sigel E. Baur R. Malherbe P. (1991). Activation of protein kinase C results in down-modulation of different recombinant GABAA-channels. *FEBS Lett.* 291 150–152 10.1016/0014-5793(91)81124-Q 1657635

[B68] Tang X. Hernandez C. C. Macdonald R. L. (2010). Modulation of spontaneous and GABA-evoked tonic alpha4beta3delta and alpha4beta3gamma2L GABAA receptor currents by protein kinase A. *J. Neurophysiol.* 103 1007–1019 10.1152/jn.00801.2009 19939957 PMC2822691

[B69] Tao W. Higgs M. H. Spain W. J. Ransom C. B. (2013). Postsynaptic GABAB receptors enhance extrasynaptic GABAA receptor function in dentate gyrus granule cells. *J. Neurosci.* 33 3738–3743 10.1523/JNEUROSCI.4829-12.2013 23447585 PMC6619291

[B70] Uusi-Oukari M. Kontturi L.-S. Coffey E. T. Kallinen S. A. (2010). AMPAR signaling mediating GABA(A)R delta subunit up-regulation in cultured mouse cerebellar granule cells. *Neurochem. Int.* 57 136–142 10.1016/j.neuint.2010.05.005 20470842

[B71] Verdoorn T. A. Draguhn A. Ymer S. Seeburg P. H. Sakmann B. (1990). Functional properties of recombinant rat GABAA receptors depend upon subunit composition. *Neuron* 4 919–928 10.1016/0896-6273(90)90145-6 1694446

[B72] Wall M. J. Usowicz M. M. (1997). Development of action potential-dependent and independent spontaneous GABA A receptor-mediated currents in granule cells of postnatal rat cerebellum. *Eur. J. Neurosci.* 9 533–548 10.1111/j.1460-9568.1997.tb01630.x 9104595

[B73] Wang D.-S. Zurek A. A. Lecker I. Yu J. Abramian A. M. Avramescu S. (2012). Memory deficits induced by inflammation are regulated by α5-subunit-containing GABAA receptors. *Cell Rep.* 2 488–496 10.1016/j.celrep.2012.08.022 22999935 PMC4391624

[B74] Wang R. A. Cheng G. Kolaj M. Randiæ M. (1995). Alpha-subunit of calcium/calmodulin-dependent protein kinase II enhances gamma-aminobutyric acid and inhibitory synaptic responses of rat neurons *in vitro*. *J. Neurophysiol.* 73 2099–2106 7623101 10.1152/jn.1995.73.5.2099

[B75] West A. H. Stock A. M. (2001). Histidine kinases and response regulator proteins in two-component signaling systems. *Trends Biochem. Sci.* 26 369–376 10.1016/S0968-0004(01)01852-7 11406410

[B76] Wisden W. Laurie D. J. Monyer H. Seeburg P. H. (1992). The distribution of 13 GABAA receptor subunit mRNAs in the rat brain. I. Telencephalon, diencephalon, mesencephalon.* J. Neurosci.* 12 1040–1062 10.1523/JNEUROSCI.12-03-01040.1992 PMC6576059 1312131

[B77] Wlodarczyk A. I. Sylantyev S. Herd M. B. Kersanté F. Lambert J. J. Rusakov D. A. (2013). GABA-independent GABAA receptor openings maintain tonic currents. *J. Neurosci.* 33 3905–3914 10.1523/JNEUROSCI.4193-12.2013 23447601 PMC3591781

[B78] Zhang N. Wei W. Mody I. Houser C. R. (2007). Altered localization of GABA(A) receptor subunits on dentate granule cell dendrites influences tonic and phasic inhibition in a mouse model of epilepsy. *J. Neurosci.* 27 7520–7531 10.1523/JNEUROSCI.1555-07.2007 17626213 PMC6672608

